# Exploring the impact of occupational exposure: A study on cardiovascular autonomic functions of male gas station attendants in Sri Lanka

**DOI:** 10.14814/phy2.70071

**Published:** 2024-10-27

**Authors:** Tania Warnakulasuriya, Kushan Medagoda, Dulani Kottahachchi, Dunya Luke, Dilesha Wadasinghe, Prasanna Rathnayake, Janaki Ariyawansa, Tharuka Dissanayake, Pavani Sandeepani, Deepthi C. De Silva, Niranga Manjuri Devanarayana

**Affiliations:** ^1^ Department of Physiology, Faculty of Medicine University of Kelaniya Sri Lanka

**Keywords:** air pollution, cardiovascular autonomic functions, fuel vapor exposure, heart rate variability, occupation health

## Abstract

Fuel dispensing at fuel stations is performed manually by unprotected male gas station attendants in Sri Lanka, who have long working hours. These workers are exposed to hydrocarbon fuels associated with multiple health effects by modulation of the autonomic nervous system. This study was performed to determine cardiovascular autonomic functions among fuel pump attendants in Sri Lanka. Fuel pump attendants (*n* = 50) aged between 19 and 65 years were identified for the study from seven fuel stations. They were compared with age‐ and gender‐matched controls (*n* = 46) without occupational exposure to fuel. A physical examination was performed before the autonomic function and heart rate variability (HRV) assessment. There were no significant differences in weight, height, or BMI between the study and the control populations (*p* > 0.05). Both the systolic blood pressure (SBP) (Mann Whitney U (MWU) = 743.5, *p* = 0.003) and diastolic blood pressure (DBP) (MWU = 686.5, *p* = 0.001) were significantly higher among the gas station attendants compared to controls. Valsalva ratio was significantly higher among the study group (MW U = 874.00, *p* = 0.043) compared to controls. The HRV analysis showed significantly higher SDNN and SD2 (MWU = 842.00, *p* = 0.034, and MWU = 843.50, *p* = 0.035 respectively) among the gas station attendants compared to controls. The changes to the cardiovascular autonomic parameters among those exposed to fuel vapor as a gas station attendant indicate an increase in sympathetic outflow to the vessels. In the occupational setting as fuel pump attendants need periodic monitoring.

## Graphical abstract

There are changes to the cardiovascular autonomic parameters among those exposed to fuel vapor in the occupational setting as fuel pump attendants. We emphasize the importance of continuous monitoring of air pollutants in the work area and periodic health assessment of the workers to prevent occupation exposure related cardiovascular pathology.

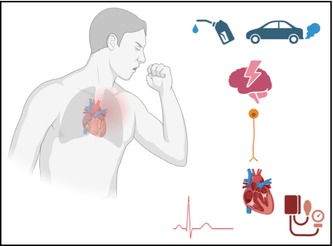

## 1 INTRODUCTION

Exposure to air pollution is associated with numerous health issues involving multiple organ systems in the human body (Bandyopadhyay, 2016; Bolden et al., 2015; Brook et al., 2004; Chuang et al., 2007; Kampa & Castanas, 2008; Manisalidis et al., 2020). In Sri Lanka, studies conducted so far have been focused on the respiratory effects of those exposed to outdoor air pollution (Nandasena et al., 2010; Senanayake et al., 2009; Thishan Dharshana & Coowanitwong, 2008). Air pollution affects the central and peripheral nervous systems (CNS and PNS) (Bandyopadhyay, 2016; Brockmeyer & D'Angiulli, 2016; Calderón‐Garcidueñas et al., 2002; Costa et al., 2020). The neurological symptoms reported among the fuel handlers exposed to volatile organic compounds (VOC) in the air include neuro‐psychosomatic symptoms, altered sleep patterns, and memory loss (Mangotra & Kumar, 2024; Thetkathuek et al., 2015).

It has been suggested that the autonomic nervous system of fuel handlers is affected by exposure to air pollutants (Chen et al., 2017; Huang et al., 2012). A few human studies have been performed to assess the alterations in autonomic nervous system functions in those chronically exposed to aromatic hydrocarbons. Attenuated cardiovascular autonomic nervous parameters, measured by heart rate variability (HRV), have been reported in these studies (Lee et al., 2011; Ma et al., 2010). Directly recorded muscle sympathetic nerve activity at rest reported increased levels with acute exposure to diesel exhausts in a recent study (Rankin et al., 2021).

When the pathophysiological mechanisms of the above are considered, direct effects of volatile gases or particulate matter on pulmonary receptors are postulated to activate neural reflexes resulting in alterations of autonomic tone (Korner, 1989). Activation of inflammatory pathways and impaired vascular functions, additionally contribute to altered autonomic functions, and these together are postulated to cause cardiovascular morbidity and mortality to those exposed to air pollution (Brook et al., 2004).

In Sri Lanka, the fuel pump attendants dispense fuel to individual vehicles using handheld nozzles while using minimum protective equipment. Being located on busy roads, long working hours, and residing at the stations mean that these mainly young males employed as gas station attendants are exposed to many toxic chemicals and particulate matter during their career. In a previous publication we reported that the fuel attendants in the Gampaha District of Sri Lanka are exposed to higher levels of benzene, toluene, and xylene (BTX) compared to the control population (Scheepers et al., 2019) and it has an effect on the spirometry parameters which correlated to the BTX exposure not only in the fuel handlers but also in controls in a distinctly different pattern (Warnakulasuriya et al., 2023). In this background, we hypothesized that the chronic exposure to fuel vapor by fuel pump attendants would alter the parameters of HRV and autonomic function assessment compared to controls.

## 2 MATERIALS AND METHODS

Male fuel station attendants (*n* = 50) from seven fuel stations in the Gampaha District aged 19–65 years were recruited as the study group. A minimum period of employment of 12 months was an inclusion criterion. Age‐ and gender‐matched controls (*n* = 50) who were not occupationally exposed to volatile hydrocarbons were recruited from the office workers residing in the Ragama *Grama Niladhari* (village officer) division. Four controls were not able to complete autonomic function (ANF) assessment adequately; hence 46 controls were selected for the final analysis.

The recruitment of participants followed verbal and written information, and signed consent. The individual findings of the assessments were made available to each of the participants.

A questionnaire was used to obtain details regarding the age, ethnicity, housing, use of motorized vehicles, exposure to environmental pollutants, and past medical history. A detailed and complete physical examination was performed by a consultant physician in General Medicine to evaluate the participants' general health. Weight and height were measured using standard techniques (Warnakulasuriya et al., 2017), and BMI was calculated.

### 2.1 Assessment of autonomic functions

Autonomic functions were assessed using a standard and validated method (Ewing et al., 1985; Low et al., 2013), and the methodology adopted in this study is briefly discussed here. A detailed description of the method used is provided in the Appendix S1 (Test procedures).

Subjects were advised to refrain from beverages containing caffeine, nicotine, or alcohol for at least 8 h before testing. Medications with adrenergic or cholinergic properties were discontinued for at least 48 h before the study, which was not needed for any of the participants. The participants attended testing at the autonomic function testing lab of the Faculty of Medicine, University of Kelaniya in the morning after 3 days of continuous shift work.

Testing was performed in thermo‐neutral conditions between 9.00 am and 11.00 am on all subjects. Subjects were allowed to acclimatize to the experimental and environmental conditions for 30 min. The testing was performed and recorded by a single observer to eliminate bias. Power lab 4/26 model number PL2604 (AD instrument) digital data recorder and analyzed using LabChart Pro software were used for testing.

The parasympathetic function was evaluated by (Choudhary et al., 2017),

1. Immediate heart rate response to standing and tilt test—The table was tilted to 70^0^ head up position within 30 s while continuously recording the ECG and BP recordings at 1, 2, 4, 7, and 10 min.
2. Heart rate variation during deep breathing—Breathing was modulated at six breaths per minute for 1 min with continuous ECG recording to obtain three consecutive artifact‐free cycles of deep inspiration and expiration.
3. Heart rate response to the Valsalva maneuver—The subject was asked to exhale into a mouthpiece connected to a mercury manometer and to maintain the expiratory pressure at 40 mmHg for 15 s while recording the ECG during and 45 s after this maneuver.

The sympathetic function was evaluated by (Carter & Ray, 2009; Choudhary et al., 2017; Korhonen, 2006; Yuenyongchaiwat, 2017),

1. Fall in systolic blood pressure in response to standing and tilt test.
2. Blood pressure and heart rate response to handgrip—30% of maximum voluntary contraction up to a maximum of 5 min with blood pressure recorded at each minute.
3. Blood pressure and heart rate response to cold. The dominant hand was immersed in water at 4°C for 2 min with blood pressure recorded at each minute.
4. Blood pressure and heart rate response to mental arithmetic. The subjects were asked to perform a subtraction of 7 from 300 for 1 min. BP was measured at the end.

Classification of the abnormal cardiovagal autonomic parameters is described in the list below. (Carter & Ray, 2009; Choudhary et al., 2017; Ewing et al., 1985; Korhonen, 2006; Low et al., 2013; Yuenyongchaiwat, 2017)

**Table:** 

Tachycardia	HR >100/min
Hypertension	SBP ≥130 mmHg or DBP≥80 mmHg
Abnormal 15:30 ratio on LTS or HUT	≤1.04
Reduced pressor response to isometric exercise	Rise in DBP <16 mmHg
Exaggerated pressor response to isometric exercise	Rise in SBP ≥30 mmHg if not hypertensive; SBP ≥60 mmHg if hypertensive
Reduced pressor response mental arithmetic	Rise in SBP ≤10 mmHg
Exaggerated pressor response to mental arrhythmic	Rise in SBP ≥30 mmHg if not hypertensive; SBP ≥60 mmHg in hypertensive
Reduced E: I ratio during deep breathing	≤1.2
Reduced Valsalva ratio	≤1.2
Reduced pressor response to Cold pressor test	Rise in SBP ≤10 mmHg
Exaggerated pressor response to cold pressor test	Rise in SBP ≥30 mmHg if not hypertensive and SBP ≥60 mmHg if hypertensive

One participant did not perform the handgrip test adequately, one participant did not tolerate the cold pressor test, and one participant did not adequately perform the mental arithmetic test, all among the controls. Hyperventilation was not performed adequately by three gas station attendants and two controls. We excluded them for the analysis of each component.

### 2.2 Heart rate variability

HRV measures the sympathovagal balance at the sinoatrial level using a noninvasive technique. HRV measurements can be broadly categorized into time‐domain indices, geometric measures, and frequency domain indices (Malik, 1996; Sztajzel, 2004).

The patient was rested and allowed to acclimatize to laboratory conditions for 30 min before recording a 5‐min lead II ECG recording using the PowerLab. The HRV parameters used for the study are SDNN, RMSSD, pNN50, LF, HF, LF/HF ratio, and total power (Table S1). One participant had ectopic beats; hence we excluded him from HRV analysis, allowing us to analyze the HRV in only 49 gas station attendants. A detailed description of the HRV parameters used is provided in the Appendix S1 (HRV parameters used in the analysis).

For the statistical analysis, nonparametric methods were used to analyze data as the age and autonomic parameters were not normally distributed as seen by the Kolmogorov–Smirnov test (*p* ≤ 0.002). Mann Whitney U (MWU) was used to compare the medians of the two populations. Nonparametric chi‐square was used to analyze categorical variables between the two groups. Spearman correlation was used to measure the association between two continuous variables. Smoking was controlled for using Quade Non parametric ANCOVA. All statistical analysis was done using SPSS/PC version 28.0.1.0 (SPSSInc., Chicago, IL), and *p* values <0.05 were considered significant.

## 3 RESULTS

A detailed description of the distribution of demographic details is given in Table 1. There were no significant differences in age, weight, height, BMI, or smoking status between the gas station attendants and the control populations (*p >* 0.05). The duration of employment for gas station attendants was 8 years (2.3–14.5 years). Among the gas station attendants 68% used jackets, 18% used boots, 17% used masks, and 2% used gloves as protective wear during the shift.

**TABLE 1:** Demographic details of the study population.

	Gas station attendants	Controls	Significance
	*n =* 50	*n =* 46	*p*
Age (Years)	38.0 (27.7–48.5)	38.5 (27.0–43.7)	0.452
Weight (kg)	64.8 (58.8–77.2)	65.45 (59.8–72.8)	0.705
Height (m)	166.0 (162.0–170.0)	166.3 (162.5–170.0)	0.985
BMI (kg/m^2^)	24.2 (20.9–27.3)	24.1 (22.2–25.9)	0.692
Smokers (*n*) %	26 53.1%	17 37.8%	0.137
Pack years among smokers years	1.1 (0.4–1.9)	0.9 (0.4–1.6)	0.822

*Note*: Median (IQR) is given above except for smokers.

**FIGURE 1:**
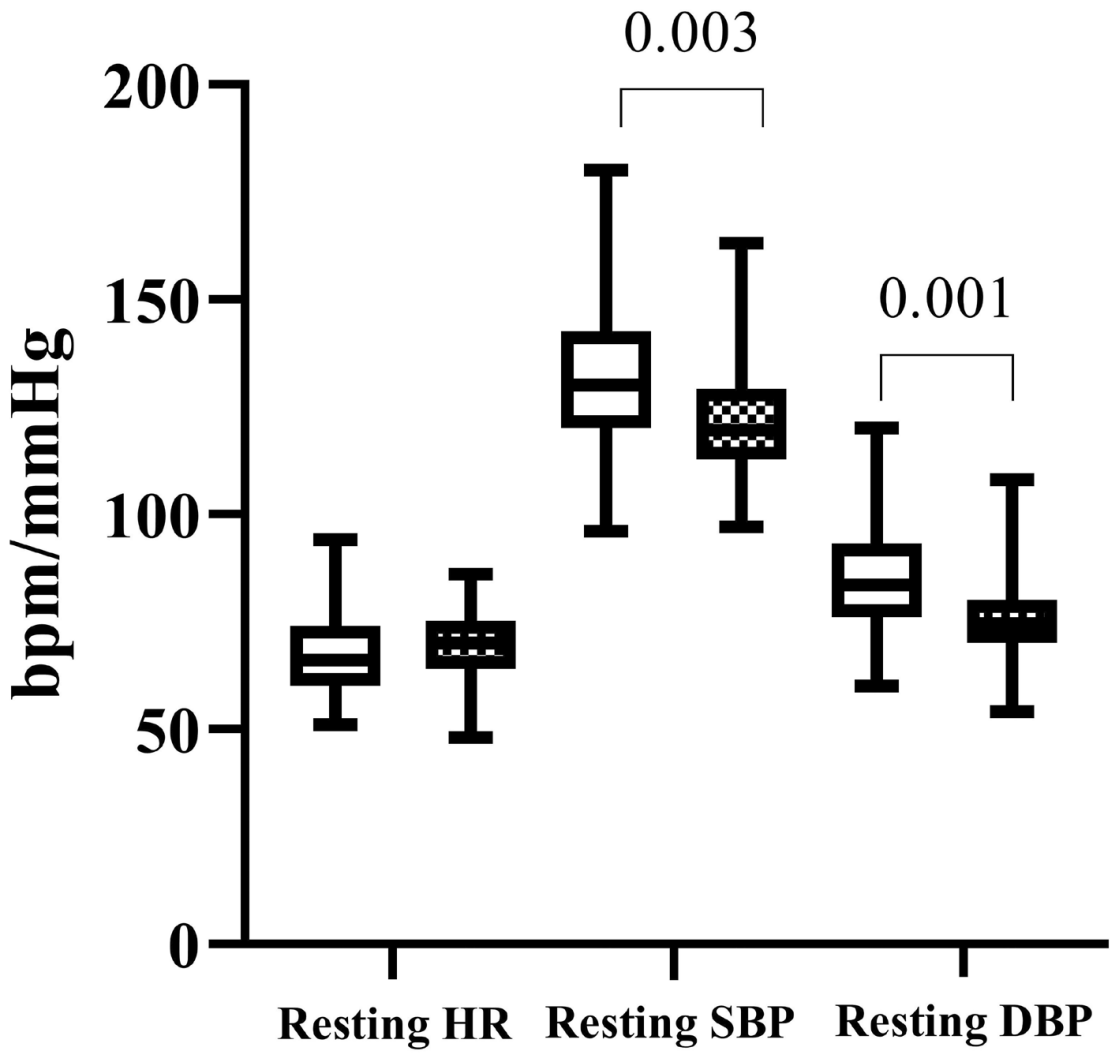
The baseline cardiovascular parameters (DBP, diastolic blood pressure; HR, heart rate; SBP, systolic blood pressure) among gas station attendants (clear box) and controls (patterned box). Lower and upper box boundaries are the 25th and 75th percentiles, respectively, with the median in between. Bars represent the 10th and 90th percentiles.

The resting heart rate was not significantly different among the study and control populations (MWU = 952.0, *p =* 0.146). However, the resting SBP was significantly higher among the fuel pump attendants (MWU = 743.5, *p =* 0.003), as was the resting DBP (MWU = 686.5, *p =* 0.001). (Figure 1 and Table 2). When controlled for smoking gas station attendants had a higher DBP (*p* = 0.002) and SD2 (*p* < 0.001).

**TABLE 2:** Baseline cardiac parameters of the study population.

	Study participants *n =* 50	Controls *n =* 46	Total *n =* 96
Resting HR bpm	66.0 (60.0–74.0)	70.0 (64.0–75.2)	69.0 (60.2–74.7)
Supine SBP^a^ mmHg	130.0 (120.0–142.5)	119.5 (112.7–129.2)	124.0 (115.2–136.7)
Supine DBP^a^ mmHg	83.5 (76.0–93.2)	74.5 (70.0–80.0)	80.0 (71.0–90.0)

^a^ Statistically significant different parameters between the study and the control populations. Median (IQR) is given in the above table.

The autonomic parameters (Table 3) show that the Valsalva ratio was significantly higher among the study group (MW U = 874.00, *p =* 0.043). A rise in DBP with sustained handgrip was significantly higher among the controls (MWU = 863.00, *p =* 0.049), but the other pressor responses were not significantly different between the two groups. The Valsalva ratio was significantly higher in the control group when controlled for smoking (*p* = 0.047) as was for DBP rise in sustained handgrip (0.048).

**TABLE 3:** Autonomic function parameters among the two populations.

Parameter	Gas station attendants	Controls	Significance MWU *p*
15:30 ratio on LTS	1.62 (1.43–2.04)	1.62 (1.40–1.85)	1069.50
			0.555
15:30 ratio on HUT	1.44 (1.31–1.71)	1.47 (1.35–1.88)	1013.50
			0.317
Delta heart rate during deep breathing	17.66 (13.75–24.37)	18.33 (14.20–24.95)	1105.00
			0.741
E:I ratio	1.34 (1.21–1.43)	1.32 (1.23–1.47)	1100.00
			0.714
Valsalva ratio	1.62 (1.34–1.76)	1.32 (1.20–1.66)	874.00
			0.043
Rise in HR in sustained handgrip (BPM)	10.00 (4.50–15.00)	32.50 (20.00–43.00)	1123.50
			0.979
Rise in SBP in sustained hand grip (mmHg)	30.00 (17.50–40.00)	32.50 (20.00–43.00)	990.50
			0.309
Rise in DBP in sustained hand grip (mmHg)	20.00 (10.00–30.50)	24.5 (16.00–40.25)	863.00
			0.049
Rise in SBP in cold pressor test (mmHg)	11.00 (3.00–20.00)	11.00 (3.75–24.25)	1053.50
			0.584
Rise in DBP in cold pressor test	57.00 (50.00–63.00)	57.50 (50.50–68.00)	1037.50
			0.505
Rise in HR in mental arithmetic (BPM)	4.00 (−0.50–10.50)	3.5 (−3.00–7.25)	997.00
			0.332
Rise in SBP in mental arithmetic (mmHg)	10.00 (1.00–20.00)	10.00 (5.75–16.25)	1122.00
			0.970
Rise in DBP in mental arithmetic (mmHg)	10.00 (3.00–15.00)	10.00 (6.00–18.00)	1027.00
			0.567
Rise in HR in hyperventilation (BPM)	22.00 (13.00–35.00)	19.00 (8.50–27.50)	817.50
			0.085

*Note*: Median (IQR) is given in the above table.

Persons with blood pressure meeting the BP criteria for diagnosis of hypertension was significantly higher among the gas station attendants compared to controls (*p =* 0.038). A significantly higher number of controls could not evoke an adequate diastolic pressor response to isometric handgrip (*p =* 0.003). An abnormal 30:15 ratio on lying to standing (LTS) was not observed in gas station attendants; one person among the controls had a 30:15 ratio below 1.04 on the head‐up tilt (HUT) (Table 4).

**TABLE 4:** Autonomic function abnormalities among the two populations.

	Gas station attendants *n =* 50	Controls *n =* 46	Significance *p*
Hypertension SBP≥130 mmHg or DBP≥80 mmHg [*N* (%)]	22 (44.0)	11 (23.9)	0.038
Reduced pressor response to Isometric exercise DBP ≤16 mmHg [*N* (%)]	23 (46.0)	8 (17.4)	0.003
Exaggerated pressor response to Isometric exercise SBP≥30 mmHg if not hypertensive and SBP≥60 mmHg in hypertensive [*N* (%)]	16 (32.0)	19 (41.3)	0.34
Reduced pressor response Mental Arrhythmic Δ SBP≤10 mmHg [*N* (%)]	20 (40.0)	19 (41.3)	0.961
Exaggerated pressor response to Mental Arrhythmic [*N* (%)]	16 (32.0)	19 (41.3)	0.34
Abnormal Deep breathing E:I ratio ≤1.2 [*N* (%)]	11 (22.0)	5 (10.9)	0.218
Abnormal Valsalva ratio ≤1.2 [*N* (%)]	11 (22.0)	5 (10.9)	0.68
Reduced pressor response to Cold pressor test Δ SBP ≤10 mmHg [*N* (%)]	14 (28.0)	13 (28.2)	0.97
Exaggerated pressor response to cold pressor test [*N* (%)]	16 (32.0)	19 (41.3)	0.34

Orthostatic hypotension was reported in similar percentages among gas station attendants (36.5%) and the controls (37.5%). Postural Orthostatic Tachycardia Syndrome (POTS) was seen among two controls, but it was not seen among gas station attendants (Table S2).

The HRV parameters using SDNN and SD2 (Table 5) were found to be significantly higher among the gas station attendants compared to controls (SDNN MWU = 842.00, *p =* 0.034; SD2 MWU = 843.50, *p =* 0.035). HRV parameters were controlled for smoking gas station attendants had a higher SDNN (*p* = 0.044) and SD2 (*p* = 0.045).

**TABLE 5:** Heart rate variability parameters among the study population.

HRV parameter	Gas station attendants *n =* 49	Controls *n =* 46	MWU *p*
SDNN ms	57.67 (42.14–74.84)	47.05 (35.31–55.82)	842.00
			0.034
RMSSD ms	36.39 (25.62–53.51)	33.43 (20.16–47.57)	941.50
			0.167
PNN50%	16.10 (4.05–31.37)	9.69 (1.23–17.19)	906.00
			0.100
Total power ms^2^	2479.00 (1355.50–5137.50)	1773.50 (996.37–2859.75)	900.00
			0.091
LF power ms^2^	711.40 (308.65–1187.50)	501.00 (271.22–871.32)	902.50
			0.095
HF power ms^2^	602.00 (240.00–1201.00)	532.30 (157.95–786.00)	1025.00
			0.447
LF/HF ratio ms^2^	1.41 (0.79–1.88)	1.32 (0.64–1.61)	1029.00
			0.466
SD1	25.77 (18.14–37.87)	23.66 (14.29–33.65)	943.00
			0.171
SD2	75.75 (54.71–98.56)	62.63 (44.97–76.72)	843.50
			0.035

*Note*: Median (IQR) is given in the above table.

Age correlated positively with the SBP and DBP but correlated negatively with Δ HR during deep breathing (DB), E:I ratio, and Valsalva ratio in both the gas station attendants and the control groups. Age correlated with 30:15 ratios among the controls in both LTS and HUT. Among the gas station attendants, hours of employment per week correlated negatively with the resting DBP (*r* = −0.293, *p* = 0.039) and positively with the ΔHR during DB (*r* = 0.399, *p* = 0.016). None of the cardiovascular autonomic parameters correlated with the duration of employment as a gas station attendant (Table S3).

BMI did not correlate with the resting HR (correlation coefficient (*r*) = 0.233, *p* = 0.104) but the BMI correlated positively with the SBP (*r* = 0.299, *p* = 0.035) and DBP (*r* = 0.324, *p* = 0.022) among the gas station attendants and in the total study population. Among the controls, BMI correlated positively with the resting HR (*r* = 0.309, *p* = 0.037), SBP (*r* = 0.508, *p* < 0.001), and DBP (*r* = 0.370, *p* = 0.011).

**FIGURE 2:**
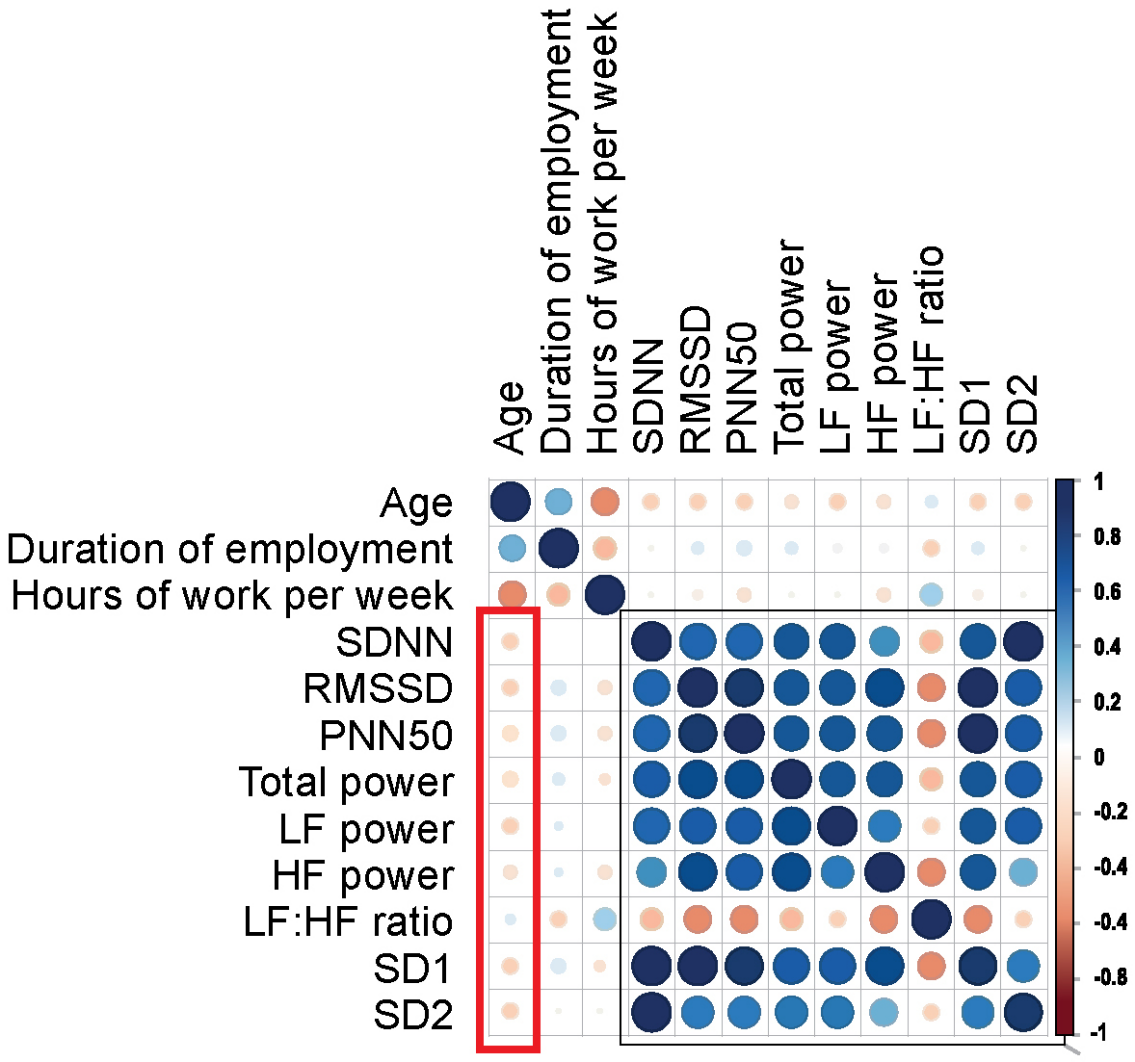
The correlation matrix shows the correlation of the age, duration of employment, and hours of exposure per week to the HRV parameters. The HRV parameters correlated negatively with age, except the LF/HF. Except for LF:HF ratio, all the other HRV parameters strongly correlated positively with each other, LF:HF ratio correlated negatively.

The LF/HF ratio correlated positively at a significant level with the hours of work per week among the gas station attendants (*r* = 0.358, *p* = 0.011), although not very strongly (Figure 2, Table S3).

## 4 DISCUSSION

This is the first study to our knowledge reporting HRV and ANF assessments in gas station attendants in Sri Lanka. A higher resting SBP and DBP among workers exposed to VOC than controls (the body weight and BMI were well matched for the subjects and controls) was observed. Among the cardio‐vagal autonomic functions assessed in this study, the Valsalva ratio, a parasympathetic measure, was significantly higher among the gas station attendants than the controls. DBP rise during handgrip, a sympathetic parameter, was significantly higher among the controls than the gas station attendants (*p =* 0.049) (Table 3). During the handgrip test, a higher number (*n =* 23, 24%) of gas station attendants were unable to evoke an adequate pressor response (≥16 mmHg) compared to only eight persons (8.3%) in the control group (*p* = 0.003, Table 4). The above results point toward an increase in sympathetic outflow to the vessels and a predominance of vagal parasympathetic influence to the control of the heart rate. HRV parameters confirm our ideation as SDNN is more influenced by the parasympathetic influence of RSA in short‐term recordings as we report here. An increase in SDNN among gas station attendants (*p* = 0.034, Table 5) indicates a vagal predominance as seen by the higher Valsalva ratio. SD2, (a predictor of baroreflex sensitivity) and LF power of HRV, is also higher in the gas station attendants, pointing toward a higher vagal response to pressure related changes via the baro‐reflex.

The interpretation of the delta BP changes with evoked responses should be done with caution as these findings could also be attributed to the exaggerated resting sympathetic stimulation (Rankin et al., 2021) to the vessels, resulting in higher resting SBP and DBP among the gas station attendants. The higher sympathetic tone would impair the response to additional sympathetic stimulation during isometric exercise testing (Shah et al., 2019). Resulting in more persons among the fuel attendants being unable to evoke an adequate pressor response for the hand grip test and had a higher maximum SBP and DBP rise for the cold pressor test and mental arithmetic (Table S4). These results indicate altered sympathetic stimulation via neural mechanisms among the gas station attendants exposed to inhaled fuel emissions.

We reported that the gas station attendants in Sri Lanka were exposed to significantly higher levels of BTX than controls (*p <* 0.01) who were office workers. The pre‐shift‐post‐shift difference in BTX levels was also significantly higher among gas station attendants (*p <* 0.01) (Scheepers et al., 2019). Neurological dysfunctions have been published among those exposed to VOC. Chronic exposure to toluene was noted to cause irreversible cerebellar, brainstem, and pyramidal tract dysfunction (Svensson et al., 1992). Low dose is chronic exposure to toluene is associated with CNS activation, while higher exposures are associated with CNS depression (Davidson et al., 2021; Guo et al., 2022; Ritchie et al., 2001; Rooseboom et al., 2023). Low dose, chronic xylene exposure in humans is associated with dizziness, reduced short‐term memory, and increased reaction time, while accidental, high dose exposure causes epileptic seizures, amnesia, cerebral hemorrhages, and loss of consciousness (Geraldino et al., 2021). Other reported effects include increased dopamine and noradrenaline secretion in the hypothalamus and median eminence (Ritchie et al., 2001). With all these CNS involvement it is inevitable that the ANS is also affected by these toxic exposures.

Autonomic nervous system dysfunction is suggested to cause cardiovascular disturbances (Bont et al., 2022; Huang et al., 2012; Riggs et al., 2022). Cardiac morbidity and mortality have been associated with air pollution (Brook et al., 2004; Zaky et al., 2015) A rise in oxidative stress and coagulation factors has also been postulated as other mechanisms increasing cardiovascular risk on exposure to air pollution (Chuang et al., 2007). In a previous study, an increase in SBP (but not DBP or HR) on exposure to diesel fuel has been reported (Cosselman et al., 2012). These authors suggested that an increase in sympathetic tone to vessels. This was directly shown by an increase to muscle SNA with acute diesel exhaust exposure (Rankin et al., 2021).

Most other studies investigating autonomic nervous system function among those exposed to fuel or other VOC have relied upon the HRV (Ji et al., 2023; Ma et al., 2010; Pope et al., 2004; Rankin et al., 2021; Weichenthal et al., 2012). Higher HRV is considered to be protective as it reflects the ability of the heart to respond to changing environmental conditions (Perez et al., 2015). Factors that influence the HRV physiologically include age, gender, circadian rhythm, and body position. Reduced HRV when these factors are accounted for suggests an increase in sympathetic tone and is associated with increased mortality among those with cardiac disease (Hillebrand et al., 2013).

In our study, we found that the global HRV measured by SDNN was significantly higher among the gas station attendants than the controls (*p =* 0.034) as was the SD2 (*p =* 0.035). All the other HRV parameters were comparable between the two groups in our study. HRV alteration points toward a vagal predominance to the heart. This could suggest either that the high blood pressure in the gas station attendants is causing a shift toward vagal stimulation via baro reflex mechanisms causing a parasympathetic predominance to the heart compared to the controls and it has been nominated as a mechanism by Rankin et al. where they recorded HRV with acute exposure to diesel exhausts (Rankin et al., 2021), The point of contention here in a setting of occupational exposure would be that the baroreflex mechanism resets at the higher blood pressure with chronic hypertension (Korner, 1989) The other possibility is that the central activation of the autonomic control centers (both sympathetic and parasympathetic) (Bont et al., 2022). The sympathetic nervous system which is differentially controlled (Pagani et al., 1974) is seen to be stimulatory to the vessels; increasing peripheral vascular resistance with fuel vapor exposure. Interestingly parasympathetic efferents to the heart may also be stimulatory (with out the baro‐receptor reflex being involved). Parallel activation of both of these systems have been reported previously in different experimental studies in both physiological and pathological conditions (Bont et al., 2022; Cerati & Schwartz, 1991; Pagani et al., 1974). We hope future studies would unravel the mechanisms through central nervous system examination in animal models or in humans via advances imaging modalities.

In an animal model, rats exposed to diesel exhausts compared with those exposed to filtered air had increased HRV parameters (triangular index and pNN15) (Carll et al., 2013). The opposite effect on HRV parameters were reported by Pope et al. in humans (Pope et al., 2004). These differences in HRV may at least partly be accounted for by the complex mixture of vehicle emissions as some components are associated with a reduction in these HRV parameters (Weichenthal et al., 2012) a single VOC exposure may cause erroneous conclusions as each component may contribute differently when inhaled in a mixture.

Much of the literature on VOC exposure has concentrated on the effects of a single component of hydrocarbon fuels (Katukam et al., 2012; Su et al., 2014; Svensson et al., 1992). Still, the complex mixture of at least 150 chemical components that constitute a single type of fuel and their additive, antagonistic and synergistic actions have not been considered. The current study we think addresses this as well.

Smoking is an important coexposure that affects the cardiovascular system and autonomic functions (Dimitriadis et al., 2022), and we controlled for smoking statistically and found the relationships to be maintained. This could have resulted from gas station attendants not smoking during the working hours and the two populations not being statistically different in the smoking status or the pack years.

In conclusion, there are significant changes to the cardiovascular autonomic parameters among those exposed to fuel vapor in the occupational setting as gas station attendants. The changes to the cardiovascular autonomic parameters among gas station attendant indicate an increase in sympathetic outflow to the vessels and the chronotropic control of the heart. Periodic health assessment of gas station attendants should be made mandatory until self service centers can be implemented eliminating the occupational exposure while pumping fuel. But petroleum refineries continue to expose persons to these fumes. Thus, we would like to advocate for more sustainable sources of energy to power locomotives.

### 4.1 Limitations

We have focused on the exposure to hydrocarbons via inhalation but it's possible that they may have had skin exposures causing a higher level expose to hydrocarbons. In a preliminary analysis, we reported an increase in the exposures with spills on some days and not on others (Scheepers et al., 2019). This is possibly due to the variability in exposure intensity and behavioral factors that may be associated with dermal exposures.

Smoking status was not matched between the two groups, we recommend matching for smoking in future studies. The higher prevalence of orthostatic hypotension in both groups (36.5% and 37.5% among gas station attendants and the controls, respectively) could be attributed to lower hydration levels among the participants on the day of testing. They were advised to fast overnight until a standard meal was given as breakfast at the laboratory. The level of hydration may have been affected by the fasting status, although they were advised to continue clear liquid intake. Hydration plays an essential role in orthostatic intolerance, and this should be given attention in future studies.

The exposure values of individual VOC components were not compared in the current publication due to the limited number of samples available. Particulate matter exposure and exposure to other harmful gasses such as NO_x_ were not assessed in this study due to restrictions in expertise and funding. For future studies, we propose that physical activity levels be assessed by objective methods (e.g., 6‐min walk test/Harvard stool test). We hope to include the assessment in future studies.

## AUTHOR CONTRIBUTIONS

TW, KM, DK, DL, DW, DDS, and NMD were collaborators in designing and carrying out the research, data analysis, and drafting the manuscript. KM, DK, DW, DL, and DDS were the physicians involved in examining the patients and data acquisition and assisted in drafting the manuscript. TW, PR, JA, and TD carried out ANF assessment, and preparation of the manuscript. TW was responsible for the data analysis. All the authors have read and approved the final manuscript.

## FUNDING INFORMATION

University of Kelaniya Internal Research Grant: RP/03/04/03/01/2017 and RP/03/04/03/01/2019.

## CONFLICT OF INTEREST STATEMENT

The authors declare that the research was conducted in the absence of any commercial or financial relationships that could be construed as a potential conflict of interest.

## ETHICS STATEMENT

The study was approved by the ethics review committee of the Faculty of Medicine, University of Kelaniya (Ref No: P/88/02/2017).

## Supporting information


Appendix S1.

## Data Availability

The data that support the findings of this study are available on request from the corresponding author.

## References

[phy270071-bib-0001] Bandyopadhyay, A. (2016). Neurological disorders from ambient (Urban) air pollution emphasizing UFPM and PM2.5. Current Pollution Reports, 2, 203–211. 10.1007/s40726-016-0039-z

[phy270071-bib-0002] Bolden, A. L. , Kwiatkowski, C. F. , & Colborn, T. (2015). New look at BTEX: Are ambient levels a problem? Environmental Science & Technology, 49, 5261–5276. 10.1021/es505316f 25873211

[phy270071-bib-0003] Brockmeyer, S. , & D'Angiulli, A. (2016). How air pollution alters brain development: The role of neuroinflammation. Translational Neuroscience, 7, 24–30. 28123818 10.1515/tnsci-2016-0005 PMC5017593

[phy270071-bib-0004] Brook, R. D. , Franklin, B. , Cascio, W. , Hong, Y. , Howard, G. , Lipsett, M. , Luepker, R. , Mittleman, M. , Samet, J. , Smith, S. C., Jr. , Tager, I. , & Expert Panel on Population and Prevention Science of the American Heart Association . (2004). Air pollution and cardiovascular disease a statement for healthcare professionals from the expert panel on population and prevention science of the American Heart Association. Circulation, 109, 2655–2671. 15173049 10.1161/01.CIR.0000128587.30041.C8

[phy270071-bib-0005] Calderón‐Garcidueñas, L. , Azzarelli, B. , Acuna, H. , Garcia, R. , Gambling, T. M. , Osnaya, N. , Monroy, S. , DEL Tizapantzi, M. R. , Carson, J. L. , Villarreal‐Calderon, A. , & Rewcastle, B. (2002). Air pollution and brain damage. Toxicologic Pathology, 30(3), 373–389. 10.1080/01926230252929954 12051555

[phy270071-bib-0006] Carll, A. P. , Lust, R. M. , Hazari, M. S. , Perez, C. M. , Krantz, Q. T. , King, C. J. , Winsett, D. W. , Cascio, W. E. , Costa, D. L. , & Farraj, A. K. (2013). Diesel exhaust inhalation increases cardiac output, bradyarrhythmias, and parasympathetic tone in aged heart failure ‐ prone rats. Toxicological Sciences, 131(2), 583–595. 23047911 10.1093/toxsci/kfs295 PMC3937610

[phy270071-bib-0007] Carter, J. R. , & Ray, C. A. (2009). Sympathetic neural responses to mental stress: Responders, nonresponders and sex differences. American Journal of Physiology. Heart and Circulatory Physiology, 296(3), H847–H853. 10.1152/ajpheart.01234.2008 19168718 PMC2660243

[phy270071-bib-0008] Cerati, D. , & Schwartz, P. J. (1991). Single cardiac vagal fiber activity, acute myocardial ischemia, and risk for sudden death. Circulation Research, 69(5), 1389–1401. 10.1161/01.RES.69.5.1389 1934362

[phy270071-bib-0009] Chen, S. Y. , Chan, C. C. , & Su, T. C. (2017). Particulate and gaseous pollutants on inflammation, thrombosis, and autonomic imbalance in subjects at risk for cardiovascular disease. Environmental Pollution, 223, 403–408. 28159399 10.1016/j.envpol.2017.01.037

[phy270071-bib-0010] Choudhary, N. , Deepak, K. K. , Chandra, P. S. , Bhatia, S. , Sagar, R. , Jaryal, A. K. , Pandey, R. M. , & Tripathi, M. (2017). Comparison of autonomic function before and after surgical intervention in patients with temporal lobe epilepsy. Journal of Epilepsy Research, 7(2), 89–98. 10.14581/jer.17014 29344466 PMC5767494

[phy270071-bib-0011] Chuang, K. J. , Chan, C. C. , Su, T. C. , Lee, C. T. , & Tang, C. S. (2007). The effect of urban air pollution on inflammation, oxidative stress, coagulation, and autonomic dysfunction in young adults. American Journal of Respiratory and Critical Care Medicine, 176(4), 370–376. 17463411 10.1164/rccm.200611-1627OC

[phy270071-bib-0012] Cosselman, K. E. , Krishnan, R. M. , Oron, A. P. , Jansen, K. , Peretz, A. , Sullivan, J. H. , Larson, T. V. , & Kaufman, J. D. (2012). Blood pressure response to controlled diesel exhaust exposure in human subjects. Hypertension, 59(5), 943–948. 22431582 10.1161/HYPERTENSIONAHA.111.186593 PMC3654814

[phy270071-bib-0013] Costa, L. G. , Cole, T. B. , Dao, K. , Chang, Y. , Coburn, J. , & Garrick, J. M. (2020). Effects of air pollution on the nervous system and its possible role in neurodevelopmental and neurodegenerative disorders. Pharmacology & Therapeutics, 210, 107523. 10.1016/j.pharmthera.2020.107523 32165138 PMC7245732

[phy270071-bib-0014] Davidson, C. J. , Hannigan, J. H. , & Bowen, S. E. (2021). Effects of inhaled combined benzene, toluene, ethylbenzene, and xylenes (BTEX): Toward an environmental exposure model. Environmental Toxicology and Pharmacology, 81, 103518. 10.1016/j.etap.2020.103518 33132182

[phy270071-bib-0015] de Bont, J. , Jaganathan, S. , Dahlquist, M. , Persson, Å. , Stafoggia, M. , & Ljungman, P. (2022). Ambient air pollution and cardiovascular diseases : An umbrella review of systematic reviews and meta‐analyses. Journal of Internal Medicine, 291, 779–800. 35138681 10.1111/joim.13467 PMC9310863

[phy270071-bib-0016] Dimitriadis, K. , Narkiewicz, K. , Leontsinis, I. , Konstantinidis, D. , Mihas, C. , Andrikou, I. , Thomopoulos, C. , Tousoulis, D. , & Tsioufis, K. (2022). Acute effects of electronic and tobacco cigarette smoking on sympathetic nerve activity and blood pressure in humans. International Journal of Environmental Research and Public Health, 19(6), 3237. 35328926 10.3390/ijerph19063237 PMC8952787

[phy270071-bib-0017] Ewing, D. J. , Martyn, C. N. , Young, R. J. , & Clarke, B. F. (1985). The value of cardiovascular autonomic function tests: 10 years experience in diabetes. Diabetes Care, 8(5), 491–498. 4053936 10.2337/diacare.8.5.491

[phy270071-bib-0018] Geraldino, B. R. , Nunes, R. F. N. , Gomes, J. B. , Poça, K. S. , Giardini, I. , Silva, P. V. B. , Souza, H. P. , Otero, U. B. , & Sarpa, M. (2021). Evaluation of exposure to toluene and xylene in Gasoline Station Workers. Advances in Preventive Medicine, 2021, 5553633. 34104483 10.1155/2021/5553633 PMC8159630

[phy270071-bib-0019] Guo, D. , Zhan, C. , Liu, J. , Wang, Z. , Cui, M. , Zhang, X. , Su, X. , Pan, L. , Deng, M. , Zhao, L. , & Liu, J. (2022). Alternations in neural oscillation related to attention network reveal influence of indoor toluene on cognition at low concentration. Indoor Air, 32(7), e13067. 35904384 10.1111/ina.13067

[phy270071-bib-0020] Hillebrand, S. , Gast, K. B. , De Mutsert, R. , Swenne, C. A. , Jukema, J. W. , Middeldorp, S. , Rosendaal, F. R. , & Dekkers, O. M. (2013). Heart rate variability and first cardiovascular event in populations without known cardiovascular disease: Meta‐analysis and dose‐response meta‐regression. Europace, 15(5), 742–749. 23370966 10.1093/europace/eus341

[phy270071-bib-0021] Huang, W. , Zhu, T. , Pan, X. , Hu, M. , Lu, S. E. , Lin, Y. , Wang, T. , Zhang, Y. , & Tang, X. (2012). Air pollution and autonomic and vascular dysfunction in patients with cardiovascular disease: Interactions of systemic inflammation, overweight, and gender. American Journal of Epidemiology, 176(2), 117–126. 22763390 10.1093/aje/kwr511 PMC3493195

[phy270071-bib-0022] Ji, X. Z. , Liu, S. , Wang, W. Z. , Zhao, Y. T. , Li, L. Y. , Zhang, W. L. , Shen, G. F. , Deng, F. R. , & Guo, X. B. (2023). Associations between indoor volatile organic compounds and nocturnal heart rate variability of young female adults: A panel study. Journal of Peking University Health Science, 55(3), 488–494. 10.19723/j.issn.1671-167X.2023.03.015 PMC10258045 37291925

[phy270071-bib-0023] Kampa, M. , & Castanas, E. (2008). Human health effects of air pollution. Environmental Pollution, 151, 362–367. 17646040 10.1016/j.envpol.2007.06.012

[phy270071-bib-0024] Katukam, V. , Kulakarni, M. , Syed, R. , Alharbi, K. , & Naik, J. (2012). Effect of benzene exposure on fertility of male workers employed in bulk drug industries. Genetic Testing and Molecular Biomarkers, 16(6), 592–597. 22304538 10.1089/gtmb.2011.0241

[phy270071-bib-0025] Korhonen, I. (2006). Blood pressure and heart rate responses in men exposed to arm and leg cold pressor tests and whole‐body cold exposure. International Journal of Circumpolar Health, 65(2), 178–184. 10.3402/ijch.v65i2.18090 16711469

[phy270071-bib-0026] Korner, P. I. (1989). Baroreceptor resetting and other determinants of baroreflex properties in hypertension. Clinical and Experimental Pharmacology and Physiology, 16(s15), 45–64. 10.1111/j.1440-1681.1989.tb02995.x 2680189

[phy270071-bib-0027] Lee, M. S. , Magari, S. , & Christiani, D. C. (2011). Cardiac autonomic dysfunction from occupational exposure to polycyclic aromatic hydrocarbons. Occupational and Environmental Medicine, 68(7), 474–478. 21172795 10.1136/oem.2010.055681 PMC3686498

[phy270071-bib-0028] Low, P. A. , Tomalia, V. A. , & Park, K. J. (2013). Autonomic function tests: Some clinical applications. Journal of Clinical Neurology, 9(1), 1–8. 23346153 10.3988/jcn.2013.9.1.1 PMC3543903

[phy270071-bib-0029] Ma, C. M. , Lin, L. Y. , Chen, H. W. , Huang, L. C. , Li, J. F. , & Chuang, K. J. (2010). Volatile organic compounds exposure and cardiovascular effects in hair salons. Occupational Medicine, 60(8), 624–630. 10.1093/occmed/kqq128 20819803

[phy270071-bib-0030] Malik, M. (1996). Heart rate variability: Standards of measurement, physiological interpretation, and clinical use. Circulation, 93(5), 1043–1065. 8598068

[phy270071-bib-0031] Mangotra, A. , & Kumar, S. (2024). Volatile organic compounds : A threat to the environment and health hazards to living organisms – A review. Journal of Biotechnology, 382, 51–69. 10.1016/j.jbiotec.2023.12.013 38242502

[phy270071-bib-0032] Manisalidis, I. , Stavropoulou, E. , & Stavropoulos, A. (2020). Environmental and health impacts of air pollution: A review. Frontiers in Public Health, 8(February), 1–13. 32154200 10.3389/fpubh.2020.00014 PMC7044178

[phy270071-bib-0033] Nandasena, Y. L. S. , Wickremasinghe, A. R. , & Sathiakumar, N. (2010). Air pollution and health in Sri Lanka: A review of epidemiologic studies. BMC Public Health, 10(1), 300. 20515506 10.1186/1471-2458-10-300 PMC2893095

[phy270071-bib-0034] Pagani, M. , Schwartz, P. J. , Banks, R. , Lombardi, F. , & Malliani, A. (1974). Reflex responses of sympathetic preganglionic neurones initiated by different cardiovascular receptors in spinal animals. Brain Research, 68(2), 215–225. 4826896 10.1016/0006-8993(74)90391-6

[phy270071-bib-0035] Perez, C. M. , Hazari, M. S. , & Farraj, A. K. (2015). Role of autonomic reflex arcs in cardiovascular responses to air pollution exposure. Cardiovascular Toxicology, 15(1), 69–78. 25123706 10.1007/s12012-014-9272-0 PMC4766835

[phy270071-bib-0036] Pope, C. A. , Hansen, M. L. , Long, R. W. , Nielsen, K. R. , Eatough, N. L. , Wilson, W. E. , & Eatough, D. J. (2004). Ambient particulate air pollution, heart rate variability, and blood markers of inflammation in a panel of elderly subjects. Environmental Health Perspectives, 112(3), 339–345. 14998750 10.1289/ehp.6588 PMC1241864

[phy270071-bib-0037] Rankin, G. D. , Kabéle, M. , Brown, R. , Macefield, V. G. , Sandström, T. , & Bosson, J. A. (2021). Acute exposure to diesel exhaust increases muscle sympathetic nerve activity in humans. Journal of the American Heart Association, 10(10), e018448. 33942621 10.1161/JAHA.120.018448 PMC8200707

[phy270071-bib-0038] Riggs, D. W. , Malovichko, M. V. , Gao, H. , McGraw, K. E. , Taylor, B. S. , Krivokhizhina, T. , Rai, S. N. , Keith, R. J. , Bhatnagar, A. , & Srivastava, S. (2022). Environmental exposure to volatile organic compounds is associated with endothelial injury. Toxicology and Applied Pharmacology, 437, 115877. 35045333 10.1016/j.taap.2022.115877 PMC10045232

[phy270071-bib-0039] Ritchie, G. D. , Still, K. R. , Alexander, W. K. , Nordholm, A. F. , Wilson, C. L. , Rossi, J., 3rd , & Mattie, D. R. (2001). A review of the neurotoxicity risk of selected hydrocarbon fuels. Journal of Toxicology and Environmental Health Part B: Critical Reviews, 4(3), 223–312. 11503417 10.1080/109374001301419728

[phy270071-bib-0040] Rooseboom, M. , Aygun, N. , North, C. , James, R. , & Segal, L. (2023). Recommendation for an occupational exposure limit for toluene. Regulatory Toxicology and Pharmacology, 141(April), 105387. 10.1016/j.yrtph.2023.105387 37169161

[phy270071-bib-0041] Scheepers, P. T. J. , de Werdt, L. , van Dael, M. , Anzion, R. , Vanoirbeek, J. , Duca, R. C. , Creta, M. , Godderis, L. , Warnakulasuriya, D. T. D. , & Devanarayana, N. M. (2019). Assessment of exposure of gas station attendants in Sri Lanka to benzene, toluene and xylenes. Environmental Research, 178(August), 108670. 10.1016/j.envres.2019.108670 31472361

[phy270071-bib-0042] Senanayake, M. P. , Samarakkody, R. P. , Sumanasena, S. P. , Kudalugodaarachchi, J. , Jasinghe, S. R. , & Hettiarachchi, A. P. (2009). A relational analysis of acute wheezing and air pollution, Sri Lanka. Journal of Environmental Science and Health, Part A, 30(3), 66.

[phy270071-bib-0043] Shah, H. , Ranjan, S. , & Karna, S. (2019). Blood pressure reactivity after isometric handgrip test in hypertensive Indian adults. Journal of Hypertension, 37, e195.

[phy270071-bib-0044] Su, J. , Li, Q. , Liang, G. , Zhang, L. , Qing, L. , & Liang, L. (2014). Effects of occupational exposure to low concentration of benzene series on serum oxidative stress levels in gas station workers. Journal of Environmental Health, 31, 242–244.

[phy270071-bib-0045] Svensson, B. G. , Nise, G. , Erfurth, E. M. , & Olsson, H. (1992). Neuroendocrine effects in printing workers exposed to toluene. British Journal of Industrial Medicine, 49(6), 402–408. 1606026 10.1136/oem.49.6.402 PMC1012121

[phy270071-bib-0046] Sztajzel, J. (2004). Heart rate variability: A noninvasive electrocardiographic method to measure the autonomic nervous system. Swiss Medical Weekly, 134, 514–522. 15517504 10.4414/smw.2004.10321

[phy270071-bib-0047] Thetkathuek, A. , Jaidee, W. , Saowakhontha, S. , & Ekburanawat, W. (2015). Neuropsychological symptoms among workers exposed to toluene and xylene in two paint manufacturing factories in eastern Thailand. Advances in Preventive Medicine, 2015, 1–10. 10.1155/2015/183728 PMC4531158 26290757

[phy270071-bib-0048] Thishan Dharshana, K. G. , & Coowanitwong, N. (2008). Ambient PM10 and respiratory illnesses in Colombo City, Sri Lanka. Journal of Environmental Science and Health, Part A, 43(9), 1064–1070. 10.1080/10934520802060035 18569321

[phy270071-bib-0049] Warnakulasuriya, D. T. D. , Peries, P. P. U. C. , Rathnasekara, Y. A. C. , Jayawardena, K. T. M. , Upasena, A. , & Wickremasinghe, A. R. (2017). Ultrasonographic parameters of the liver, spleen and kidneys among a cohort of school children in Sri Lanka. BMC Pediatrics, 17(1), 192. 10.1186/s12887-017-0943-4 29145822 PMC5692795

[phy270071-bib-0050] Warnakulasuriya, T. , Medagoda, K. , Amarasiri, L. , Wadasighe, D. , Kottahachchi, D. , Ariyawansa, J. , Rathnayake, P. , Dissanayake, T. , Fernando, S. , & de Werdt, L. (2023). Association of Lung Function with Benzene, Toluene and Xylenes (Btx) in End‐Exhaled Air in Gas Station Attendants.

[phy270071-bib-0051] Weichenthal, S. , Kulka, R. , Bélisle, P. , Joseph, L. , Dubeau, A. , Martin, C. , Wang, D. , & Dales, R. (2012). Personal exposure to specific volatile organic compounds and acute changes in lung function and heart rate variability among urban cyclists. Environmental Research, 118, 118–123. 10.1016/j.envres.2012.06.005 22776327

[phy270071-bib-0052] Yuenyongchaiwat, K. (2017). Cardiovascular response to mental stress tests and the prediction of blood pressure. Indian Journal of Psychological Medicine, 39(4), 413–417. 10.4103/0253-7176.211744 28852231 PMC5559985

[phy270071-bib-0053] Zaky, A. , Ahmad, A. , Dell'Italia, L. J. , Jahromi, L. , Reisenberg, L. A. , Matalon, S. , & Ahmad, S. (2015). Inhaled matters of the heart. Cardiovascular Regenerative Medicine, 27, 2. 10.14800/crm.997 PMC4672864 26665179

