# Supplementary material for: Exploring the impact of occupational exposure: A study on cardiovascular autonomic functions of male gas station attendants in Sri Lanka
Source: Physiol Rep. 2024 Oct 27;12(21):e70071. doi: 10.14814/phy2.70071 (PMC11513408; doi:10.14814/phy2.70071)
Supplement: Supplementary file 1 — Appendix S1. [file PHY2-12-e70071-s001.docx]

Supplementary Material

1. **Sample size calculation**

The sample size was calculated by the software program WINPEPI using the following formula: n = (Z_α/2_+Z_β_)^2^ *2*σ^2^ / d^2^

Where, Z_1-α/2_ is the critical value of the normal distribution at 1-α/2 (for a confidence level of 95%, α is 0.05 and the critical value is 1.96), Z_1-β_ is the critical value of the normal distribution at β (for a power of 80%, β is 0.2 and the critical value for 1-β is 0.84), σ^2^ is the population variance, and d is the difference to be detected.

The following assumptions were made in the sample size calculation.

- The standard deviation between the two groups for ANF parameters were assumed to be the same with a value of 5.
- The significance level- 0.05
- The power of the study - 80%
- The difference between the mean ANF between the two groups -5

A minimum sample size of 16 persons from the exposed group and 16 controls were required (WinPepi Version 3.07). To achieve the minimum number of study population we recruited 50 gas station attendants and 50 controls as we anticipated human and technical difficulties when gathering data.

1. **Test procedures – Cardiovascular autonomic function testing.**

A more detailed description of the following tests is given in the section below.

- Resting heart rate (HR) and heart rate variability (HRV)
- HR and blood pressure (BP) response to standing and tilt test.
- HR variation with deep breathing
- Valsalva ratio
- Blood pressure and heart rate response to hand grip
- Blood pressure and heart rate response to cold
- Blood pressure and heart rate response to mental arithmetic
  1. **Resting heart rate and heart rate variability**^1^

Testing was performed after a 30-minute mandatory rest period following which ECG (lead II) recording was started along with blood pressure recordings at 5-minute intervals. The lowest blood pressure recording was used for analysis. An automated blood pressure meter (RossMax CH155), calibrated against a standard mercury sphygmomanometer (ABN Precision), was used to measure the BP. The resting heart rate was calculated from the ECG. The resting HR and HRV were recorded by Powerlab (AD instrument) (26T AD instrument) and analysed via Labchart pro software (Ver8.1.6).

- 1. **HR and blood pressure (BP) response to standing and tilt.**^2^

At the end of the rest period, the patient was asked to stand up within 3 seconds from the supine position. The ECG recordings were continued, and the BP recordings were repeated at one, three and five minutes while standing. The postural change in the BP was determined by comparison of the supine to the lowest standing BP. The ratio between the longest R-R interval (around the 30^th^ beat) to the shortest R-R interval (around the 15^th^ beat) after standing was used to determine the heart rate ratio (30:15 ratio).

The tilt test involved tilting the table to 70^0^ head up position within 30 seconds while continuously recording the ECG and BP recordings made at 1,2,4,7 and 10 minutes.

During standing or on head up tilt position thoracic blood volume is distributed to the dependent areas of the body leading to a reduction in the venous return which results in a reduction in the cardiac output, causing a baro-receptor mediated rapid increase in the heart rate which is maximum around the 15^th^ beat after standing. A vagally mediated relative overshoot bradycardia follows it, which is maximal around the 30^th^ beat. When there is an increased vagal response, the ratio is increased. An increased parasympathetic response to the manoeuvre was taken as an increase in the ratio.

- 1. **HR variation with deep breathing**^2^

This was performed in the supine position with the subject instructed to breathe deeply at six breaths per minute with continuous ECG recording to obtain three consecutive artefact-free cycles of deep inspiration and expiration. The delta heart rate (ΔHR) was taken as the mean of the difference between maximum and minimum heart rate for the six cycles. The expiration: inspiration ratio was calculated with the maximum HR at inspiration and minimum HR in expiration.

Physiologically the heart rate increases in inspiration and decreases in expiration through a vagally mediated mechanism. The origin of the heartbeat is at the sinoatrial node (SA) of the heart. At rest there is a continuous autonomic input to the SA node with vagal predominance, therefore in healthy individuals at rest, the heart rate is maintained around 60-80 beats per minute. When the subject takes a deep breath, the tidal volume expands the lungs leading to the activation of stretch receptors in the lung, the chest wall and heart chambers and hence stimulates vagal afferents. Modulation at the nucleus solitaries in the brain stem leads to a decrease in the parasympathetic tone and an increase in heart rate. The reverse happens in expiration. An increased parasympathetic response to the manoeuvre was taken as a widening of the difference.

- 1. **The Valsalva ratio**^2^

The subject was asked to exhale into a mouthpiece connected to a mercury manometer and to maintain the expiratory pressure at 40 mmHg for 15 seconds while recording the ECG during and 45 seconds after this manoeuvre. The Valsalva ratio was calculated between the maximal R-R interval (after the release of strain) and the minimal R-R interval during strain.

Forced expiration causes an initial rise in BP and a fall in HR (phase I). With the increase in thoracic pressure during blowing (overtaking the pressure of the great veins) venous return reduces and causes the BP to reduce below the baseline (early phase II). The reduction in BP causes the baroreceptors to discharge in the carotid sinus and aortic arch. The efferent vagal/parasympathetic discharge is reduced and sympathetic discharge increases resulting in a rise in HR and BP (late phase II). With the removal of forced expiration, the BP falls (phase III) and then rises to baseline (phase IV). As venous return is established a transient overshoot of BP is seen which causes a baroreceptor discharge resulting in a reduction in HR and a decline in BP to baseline.

The initial rise in BP in phase I due to mechanical effect causes the baroreceptor discharge and an initial withdrawal of vagal efferent causing the initial rise in HR and the stimulation of sympathetic discharge to continue the increase in the HR. (HR changes in phase II early and late).

- 1. **Blood pressure response to sustained handgrip**^2^

A hand grip dynamometer was used to perform maintained hand grip is at 30% of maximum voluntary contraction up to a maximum of five minutes with blood pressure recorded at each minute. The difference between the diastolic blood pressure just before the release of the hand grip and at the start was used to measure the response.

Voluntary muscle activity causes the sympathetic nervous system to be stimulated. It causes a rise in HR and BP. The metabolites of sustained exercise would also cause the sympathetic stimulation to be sustained. Total peripheral resistance will be increased due to central command activation via the sympathetic system. A rise in the diastolic BO will be caused by the above.

- 1. **Blood pressure and heart rate response to cold**^3^

The HR and BP response to cold was recorded 2 minutes after immersion of one hand in water at 4 ^0^C. The systolic blood pressure, diastolic blood pressure and heart rate were measured and compared to the resting value.

Cold causes the cold /temperature receptors and pain receptors on the skin to be stimulated. This would result in stimulation of sympathetic discharge causing elevation of HR and BP.

- 1. **Blood pressure and heart rate response to the performance of mental arithmetic** ^4^

The subjects were asked to perform a subtraction of 7 from 300 for one minute. Systolic blood pressure, diastolic blood pressure, and heart rate response compared to resting values.

Stress is induced by the instructions for calculation causing sympathetic discharge. This increases the HR and BP response.

**HRV parameters were used in the analysis.**^1^

Time domain measurements – Interval between normal successive cardiac cycles is detected i.e., heart rate change over time. The normal RR interval is determined by analysis of the QRS complexes when the heart is in sinus rhythm. Mean RR interval, the difference between the longest and the shortest RR intervals, and mean HR (and the difference between the mean daytime and nighttime heart rate in 24-hour analysis) are simple calculations. Statistical measurements are available in two forms; (1) beat-to-beat intervals or measurements derived from the intervals (SDNN, SDANN, SD) (2) instantaneous HR and measurements derived from the difference between the adjacent RR intervals (RMSSD, pNN50).

Frequency domain

Also called power spectral analysis frequency domain measures periodic oscillations of the heart rate at different frequencies and amplitudes. The variance or the power is the relative intensity of the heart’s sinus rhythm.

There are a couple of ways to obtain frequency domain analysis.

1. Non-parametric method – assessment of discrete peaks for several frequency components – Fast Fourier transformation

Individual RR intervals are transformed into bands with different spectral frequencies. By dividing these from the mean RR interval length the bands can be converted to Hertz (Hz).

1. Parametric Method – Assessment of a smooth spectrum of activity – Autoregressive model estimation

Four bands can be classified in power spectral analysis.

- Ultralow frequency band (ULF)
- Very Low-frequency band (VLF)
- Low-frequency band (LF)
- High-frequency band (HF)

Long-term recordings are needed to assess ULF components, short-term recordings of 5-10 mins suffice for the VLF, LF and HR components. The spectral component can be evaluated in terms of the frequency and the amplitude which is the area of each of the components measured as ms^2^.

Total power is the sum of all four bands (ULF, VLF, LF, HF) and is the total variance.

ULF – measures the circadian rhythm and neuroendocrine rhythms.

VLF - the long period rhythms, a marker of physical activity thus a marker of sympathetic component.

LF – modulated by both sympathetic and parasympathetic components.

HF – is a marker of vagal modulation. It is determined by the frequency of breathing.

LF/HF ratio measures sympathovagal balance. In a normal resting adult, it is between 1 and 2.

# Supplementary Figures and Tables

- 1. **Supplementary Tables**

**Supplementary Table S1**. HRV parameters used in the analysis.

| Parameter | Unit | Method of analysis | Interpretation |
| --- | --- | --- | --- |
| SDNN | ms | the standard deviation of all RR (NN) intervals | a global index of HRV |
| SD | ms | the standard deviation of differences between adjacent RR intervals | reflect day/night changes |
| RMSSD | ms | the square root of the mean of the sum of the squares of differences between adjacent RR interval | Short-term HRV changes not dependent on day/night changes, vagally mediated changes. Stable, and more suitable for clinical use. |
| pNN50 | % | per cent of the difference between adjacent RR intervals that are greater than 50 ms | Short-term HRV changes not dependent on day/night changes, vagally mediated changes, less stable and thus less suitable for clinical use. |
| LF | ms^2^ | Low-frequency power in power spectral analysis | Marker of both sympathetic and parasympathetic modulation. |
| HF | ms^2^ | High-frequency power in power spectral analysis | Marker of vagal modulation. It is determined by the frequency of breathing. |
| LF/HF ratio |  | LF/HF ratio | Measurement of sympathovagal balance. In a normal resting adult, it is between 1 and 2. |
| Total power |  | Total power is the sum of all four bands (ULF, VLF, LF, HF) in power spectral analysis | Measure the total variance. |

**Supplementary Table S2**. Orthostatic intolerance among the two populations

|  | Study participants *n*=50 | Controls *n*=46 | Significance  *p* | Total *n*=96 |
| --- | --- | --- | --- | --- |
| Orthostatic Hypotension observed at LTS |  |  |  |  |
| N | 35 | 36 | 0.48 | 71 |
| % | 36.5 | 37.5 |  | 74.0 |
| PoTs |  |  |  |  |
| N | 0 | 2 | 0.22 | 2 |
| % | 0 | 2.1 |  | 2.1 |

**Supplementary Table S3**. Correlation of HRV parameters with age, duration of employment and hours of work per week.

|  | Age | Age | Duration of employment fuel stations  Study participants *n*=49 | Hours of work at fuel stations  Study participants *n*=50 |
| --- | --- | --- | --- | --- |
|  | Study participants *n*=49 | Controls *n*=46 |  |  |
| SDNN |  |  |  |  |
| Correlation coefficient | -0.188 | 0.019 | 0.088 | -0.032 |
| *p* | 0.195 | 0.900 | 0.554 | 0.824 |
| RMSSD |  |  |  |  |
| Correlation coefficient | -0.241 | -0.092 | 0.065 | -0.033 |
| *p* | 0.095 | 0.544 | 0.663 | 0.824 |
| PNN50 |  |  |  |  |
| Correlation coefficient | -0.234 | -0.101 | 0.048 | -0.023 |
| *p* | 0.105 | 0.506 | 0.747 | 0.875 |
| Total power |  |  |  |  |
| Correlation coefficient | -0.185 | 0.066 | 0.057 | 0.018 |
| *p* | 0.202 | 0.665 | 0.700 | 0.904 |
| LF power |  |  |  |  |
| Correlation coefficient | -0.273 | 0.188 | -0.031 | 0.120 |
| *p* | 0.058 | 0.437 | 0.835 | 0.411 |
| HF power |  |  |  |  |
| Correlation coefficient | 0.285 | -0.020 | 0.041 | -0.013 |
| *p* | 0.047 | 0.893 | 0.781 | 0.929 |
| LF/HF ratio |  |  |  |  |
| Correlation coefficient | 0.081 | 0.251 | -0.260 | 0.358 |
| *p* | 0.581 | 0.092 | 0.074 | 0.011 |
| SD1 |  |  |  |  |
| Correlation coefficient | -0.243 | -0.084 | 0.063 | -0.032 |
| *p* | 0.092 | 0.581 | 0.671 | .828 |
| SD2 |  |  |  |  |
| Correlation coefficient | -0.196 | 0.053 | 0.053 | 0.085 |
| *p* | 0.176 | 0.724 | 0.719 | 0.561 |

Supplementary table S4. Maximum blood pressure reached with ANF testing in the 2 groups.

| Parameter |  | Gas station attendants | Controls | Significance  MWU  *p* |
| --- | --- | --- | --- | --- |
| Rise in SBP in Sustained hand grip (mmHg) | Median | 156.00 | 152 | 960.50  0.215 |
|  | IQR | 139.50-172.00 | 132.75-168.25 |  |
| Rise in DBP in Sustained hand grip (mmHg) | Median | 103 | 100 | 1052.00  0.576 |
|  | IQR | 89.5-119.5 | 92.75-112.25 |  |
| Rise in SBP in Cold pressor test (mmHg) | Median | 140.00 | 129.00 | 845.00  0.036 |
|  | IQR | 129.00-154.50 | 125.25-141.25 |  |
| Rise in DBP in Cold pressor test | Median | 93.00 | 88.00 | 846.00  0.036 |
|  | IQR | 84.00-100.00 | 80.00-93.00 |  |
| Rise in SBP in mental arithmetic (mmHg) | Median | 135 | 129.00 | 823.50  0.024 |
|  | IQR | 126-153.5 | 118.00-142.00 |  |
| Rise in DBP in mental arithmetic (mmHg) | Median | 90 | 89.00 | 827.00  0.037 |
|  | IQR | 86.5-104.00 | 79.00-95.50 |  |

1. **References**

Ewing, D. J., Martyn, C. N., Young, R. J., & Clarke, B. F. (1985). The Value of Cardiovascular Autonomic Function Tests: 10 Years Experience in Diabetes. *Diabetes Care*, *8*(5), 491–498. https://doi.org/10.2337/diacare.8.5.491

Hillebrand, S., Gast, K. B., De Mutsert, R., Swenne, C. A., Jukema, J. W., Middeldorp, S., … Dekkers, O. M. (2013). Heart rate variability and first cardiovascular event in populations without known cardiovascular disease: Meta-analysis and dose-response meta-regression. *Europace*, *15*(5), 742–749. https://doi.org/10.1093/europace/eus341

Low, P. A., Tomalia, V. A., & Park, K.-J. J. Autonomic function tests: Some clinical applications. , 9 Journal of Clinical Neurology (Korea) § (2013).

Malik, M. (1996). Heart rate variability: Standards of measurement, physiological interpretation, and clinical use. *Circulation*, *93*(5), 1043–1065. https://doi.org/10.1161/01.CIR.93.5.1043

Pope, C. A., Hansen, M. L., Long, R. W., Nielsen, K. R., Eatough, N. L., Wilson, W. E., & Eatough, D. J. (2004). Ambient particulate air pollution, heart rate variability, and blood markers of inflammation in a panel of elderly subjects. *Environmental Health Perspectives*, *112*(3), 339–345. https://doi.org/10.1289/ehp.6588

Sztajzel, J. (2004). Heart rate variability: A non-invasive electrocardiographic method to measure the autonomic nervous system. *Swiss Medical Weekly*, Vol. 134, pp. 514–522. https://doi.org/2004/35/smw-10321
